# Wireless Sensor Networks Fault-Tolerance Based on Graph Domination with Parallel Scatter Search

**DOI:** 10.3390/s20123509

**Published:** 2020-06-21

**Authors:** Abdel-Rahman Hedar, Shada N. Abdulaziz, Emad Mabrouk, Gamal A. El-Sayed

**Affiliations:** 1 Department of Computer Science in Jamoum, Umm Al-Qura University, Makkah 25371, Saudi Arabia; gaelsayed@uqu.edu.sa or; 2 Department of Computer Science, Faculty of Computers & Information, Assiut University, Assiut 71526, Egypt; 3 Center for Middle Eastern Studies, Lund University, SE-22362 Lund, Sweden; shada.abdulaziz@cme.lu.se; 4 Department of Mathematics, Faculty of Science, Assiut University, Assiut 71516, Egypt; mabrouk@aun.edu.eg or; 5 College of Engineering and Technology, American University of the Middle East, Kuwait, Kuwait; 6 Electrical Engineering Department, Assiut University, Assiut 71516, Egypt

**Keywords:** wireless sensor network, virtual backbone, fault-tolerance, parallel scatter search, minimum connected dominating set

## Abstract

In wireless sensor/ad hoc networks, all wireless nodes frequently flood the network channel by transmitting control messages causing “broadcast storm problem”. Thus, inspired by the physical backbone in wired networks, a Virtual Backbone (VB) in wireless sensor/ad hoc networks can help achieve efficient broadcasting. A well-known and well-researched approach for constructing virtual backbone is solving the Connected Dominating Set (CDS) problem. Furthermore, minimizing the size of the CDS is a significant research issue. We propose a new parallel scatter search algorithm with elite and featured cores for constructing a wireless sensor/ad hoc network virtual backbones based on finding minimum connected dominating sets of wireless nodes. Also, we addressed the problem of VB node/nodes failure by either deploying a previously computed VBs provided by the main pSSEF algorithm that does not contain the failed node/nodes, or by using our proposed FT-pSSEF algorithm repairing the broken VBs. Finally, as nodes in a VB incur extra load of communication and computation, this leads to faster power consumption compared to other nodes in the network. Consequently, we propose the virtual backbone scheduling algorithm SC-pSSEF which aims to find multiple VBs using the VBs provided by the pSSEF algorithm and switch between them periodically to prolong the network life time.

## 1. Introduction

Wireless sensor networks (WSNs) consist of small nodes with sensing, computation, and wireless communications capabilities. These networks are widely used in many applications such as environment monitoring, disaster forecast, battlefield detection, traffic control, and disease diagnosis. Such a wide field of applications in wireless networking motivated the interest researchers to investigate an array of wide issues related to the nature of wireless ad hoc and sensor networks [1]. Most of WSNs are distributed to monitor and control systems where human intervention is not possible or undesirable. One of the typical characteristics of WSNs is the ability to deploy many nodes in a specific area to ensure adequate coverage for this region. However, in a relatively crowded network, many problems are exacerbated such as nodes overlap with each other, nodes failure and connection loss. Moreover, a node may lose a large transmission power to unnecessarily connect to a remote base station [2]. These problems can be solved by selecting some nodes as a virtual backbone (VB) for a network. A VB is a subset of the nodes in the network where each node is either in the subset or neighbor of at least one node in the subset. Therefore, finding a virtual backbone reduces the communication overhead, increases the bandwidth efficiency, decreases the overall energy consumption. Consequently, WSN management using different VBs increases the network effective lifetime in a WSN. Computing VBs in WSNs can be reformulated as finding the Minimum Connected Dominating Set (MCDS) in graph theory [1,3].

The topology of the wireless nodes can be modeled as simple undirected graph G=(V,E), where V(G) is the set of vertices (or nodes), E(G) is the set of edges connecting the nodes of the graph. A vertex *v* of *G* is said to dominate itself and any vertex adjacent to it in *G*. A dominating set (DS) is a set of vertices which is collectively dominate all the other vertices in the graph. If the graph is connected, a connected dominating set (CDS) is a DS which is also a connected subgraph of *G*. The nodes in CDS are called dominators (backbone nodes), other nodes in the network are called dominatees (non-backbone nodes). The MCDS problem seeks to find a connected dominating set of minimum cardinality, which its size is called the domination number of *G* and written as γ(G) [4].

Scatter Search (SS) is one of the meta-heuristic search algorithms that has been successfully applied to hard optimization problems. Unlike other evolutionary methods such as genetic algorithms, SS relies on the hypothesis that systematic designs and methods for creating new solutions afford significant benefits, while the other approaches resort to using randomization. It employs strategies for search diversification and intensification that have proved effective in a wide variety of optimization problems [5,6,7].

Most basic meta-heuristic algorithms are sequential and commonly used for real-world problems emerging in both academic and industrial domains. However traditional sequential methods do not always satisfy the requirements of these real-world problems. Therefore, parallelism appears quite naturally as an effective approach not only to speed up the search by performing several computations at the same time but also to improve the quality of the provided solutions. On the other hand, the advent of multi-core processors provides a massively huge computation power for scientific and business applications. Therefore, the need for writing efficient optimized codes and data structures became more essential to achieve the expected performance and use of the computing resources [8].

The problem of a sensor node failure, missing of communication link or data, are inescapable in the WSNs. These failure problems are due to various factors such as environmental impact, battery depletion, physical damage, radio interference, etc. Fault-tolerance is the ability of a network to provide a desired level of functionality without interruption even if there are faults in the network [9]. Therefore, the fault-tolerance of a network is considered to be one of the most critical issues in WSNs. Besides the fault-tolerance, the network lifetime is another challenge faces the wireless networks that requires energy-efficient techniques to maximize the system lifetime. Although a single virtual backbone reduces the overall energy consumption of the network, the nodes in a VB have an extra load of communication and computation. This leads to consuming their energy faster than other nodes in the network. An intuitive way to solve this problem is to construct multiple VBs and switch the work between them periodically [10].

In this paper, we investigate the problem of identifying the VBs of WSNs using graph domination and scatter search in parallel environment. In addition to that we consider the problem of making such VB fault-tolerant. Finally, we investigate the problem of increasing the network life time by VB scheduling. Therefore, a new parallel Scatter Search algorithm with Elite and Featured cores (pSSEF) method is proposed for solving the minimum connected dominating set problem in wireless networks. In the pSSEF method, the population is divided among the parallel workers, so that each worker runs different scatter search methods on its solution set. Then, each worker sends its modified solution set to the elite and the featured cores, where each of them selects its solutions among the received solutions and apply special refinement procedures to improve those solutions. This algorithm achieves better results compared to other methods in the literature including the sequential method, particularly for the high dimension graph. Moreover, the algorithm is so stable as each result matches or close to the average result. To recover from (tolerate) failures that may occur in the wireless networks, we propose a new virtual backbone fault-tolerant algorithm based on scatter search called the FT-pSSEF method. The FT-pSSEF method is a modified version of the pSSEF method that keeps network working despite failure in one sensor node or more. If the failed sensor node is not among VB nodes, then it will not affect the network functionality. Otherwise, the FT-pSSEF method looks for another VB among previously obtained from the pSSEF method and kept in VB set. It picks the one that does not contain the failed node/nodes, else the FT-pSSEF method starts to look for a new set of VBs that excludes failed node/nodes. Another version of the algorithm is also designed to increase the network life time by VB scheduling called the SC-pSSEF method.

This paper is organized as follows. The next section introduces related works in dominating sets and the network virtual backbone fault-tolerance and scheduling. In Section 3, the details of the parallel proposed method are explained. The algorithmic implementations of the main proposed method to the network virtual backbone fault-tolerance and scheduling are presented in Section 4. The used benchmarks, parameter settings, the algorithmic performance analysis and comparison results are presented and discussed in Section 5. Finally, the conclusion makes up Section 6.

## 2. Related Works

A large number of network problems can be converted into graph problems. In this section, we discuss the existent work of modelling the considered network problems using graphs. Moreover, some recent studies are mentioned and discussed about the different mythologies used to solve those problems.

### 2.1. Network Models Using Graphs

Wireless sensor networks can be modeled as a graph, where a sensor node is represented as a vertex in the graph and a communication link is represented as an edge connecting two vertices. Generally, wireless sensor/ad hoc networks has no physical backbone connecting their nodes. Therefore, finding a VB of a given network is crucial in managing the network, saving communication bandwidth and sensor nodes battery, and improving routing. In recent years, it was verified that using domination in network graphs is an optimal choice to find the VBs in wireless ad hoc and wireless sensor networks [11,12,13]. The dominating and connected dominating sets have been widely used in the literature to model the topology of wireless ad hoc networks [12]. Since wireless sensor nodes have limited resources to power up, the major drawback are node/nodes failure and the lifetime of the network. Therefore, solving these problems attracted attention of researchers because the CDS can optimize the energy consumption, route data fast, and prolong the lifetime of the wireless sensor network. So, minimizing the size of the CDS which is denoted by the MCDS problem is an important research direction [4,14,15]. Since the MCDS problem is an NP-hard, there is no exact efficient algorithm available [16].

By reviewing most of existing research in managing WSNs, two essential research directions have attracted researchers: the first one pays attention to the fundamental aspects and characteristics of wireless ad hoc networks or wireless sensor networks and their application domains. Consequently, developing new algorithms to identify MCDS is an important step forward to manage such networks. Alternatively, the other research direction focuses on graph theory and solving the MCDS problem heuristically [17,18,19,20].

There are many interests in studying the MCDS problem [21,22,23,24,25]. Zhang et al. developed a centralized genetic algorithm which divides WSNs into coverage sets. They developed a localized style method based on that centralized algorithm [26]. To reduce more energy consumption, they recommended a parallel implementation version for their algorithm.

Developing other meta-heuristics to solve the MCDS problem has attracted many researchers. Li et al. designed GRASP to solve that problem [27]. Jovanovic et al. have developed ant colony optimization algorithms for the minimum connected dominating set problem (MCDS) [28]. In [29], the authors used greedy-based GA to develop two population-based algorithms for the minimum weighted connected dominating set problem. In [30], two meta-Heuristics (memetic and simulated annealing) are presented to solve the MCDS problem for wireless networks design and management.

WSNs size for most applications is huge and number of sensor nodes is enormous. Consequently, the need for more computing power steers researchers towards high performance computing and parallel processing. Hedar and El-Sayed proposed a parallel genetic algorithm with elite and diverse cores for constructing a wireless network virtual backbone based on identifying MCDS of wireless nodes to be used in wireless network topology control. Experimental results demonstrated that the proposed parallel algorithm achieved better results than their sequential version [31].

### 2.2. Virtual Backbone Network Fault-Tolerance

The WSN typically consists of hundreds or maybe thousands of small size, low cost, and low-power sensor nodes. These nodes which have limited memory, processing capability, and storage capacity, communicate with each other via a wireless medium to perform a common task. These networks are most widely used in fire prevention, industrial process control, environmental monitoring, military surveillance, etc. As WSNs are deployed in very large scale areas, the probability of sensor node failure in the network is extremely high which may result in a system disruption and thus can cause a system failure [32]. There are many reasons which may contribute to the failure problem such as environmental impact, battery depletion, physical damage, radio interference, etc. As the popularity and applications of the WSNs expand, the need to make these networks more reliable increases. This leads to the development of the fault-tolerant WSNs [9]. Fault-tolerance in WSNs is the property that a network continues providing its services despite the failure of some sensor nodes. Due to limited battery life and difficulty of recharging or replacing failure node, sensor nodes are deployed in a dense manner in the target area to increase coverage and connectivity. However, if all sensor nodes work continuously, this expedites their batteries consumption and thus affects the network lifetime. Since sensor nodes are prone to failure, WSNs applications must consider some significant features such as security, availability, fault-tolerance, and reliability, etc. Hence, fault-tolerance is one of the most recent research trends in wireless sensor networks [33].

There are two essential steps to tackle faults in the WSNs: fault detection and fault recovery. Fault detection focuses on detecting the functional failure in the network and whether it will exceed this failure in the near future or not. The second step after the fault detection is fault recovery to enable the system to overcome the detected fault. Some faults such as the depletion of battery and failure in links can be detected by a sensor node itself, this type of detection technique called self-diagnosis. Another technique is the cooperative diagnosis technique which deals with faults require cooperation between a set of sensor nodes to be diagnosed [34]. The most popular technique for fault recovery depends on replicating components that are prone to failure. In monitoring systems, a sensed data is forwarded to a base station. Thus, if some sensor nodes fail to forward the data, the base station still receives adequate data from the deployed redundant nodes in that region. Furthermore, providing multiple paths routing adds more reliability to routes if some links fail along a route [33].

Since there is no predefined or fixed infrastructure for WSNs, all sensors are frequently flooded with control messages which causes contentions, collisions, and redundancy. As a solution for this issue, the VB has been proposed as the routing infrastructure of these networks. Using VB, routing messages are only forwarded between sensors via the VB, instead of being flooded to all other sensors. Consequently, this technique reduces routing overhead and energy consumption, and prolongs the network lifetime [35]. As sensor nodes in the VB need to relay other sensors’ traffic, they are more prone to failure. Therefore, fault-tolerance enables VB to provide its specified service during a link or a node failure. Thus, fault-tolerance becomes one of the most significant research problems [36]. As the size of WSNs increases, the probability of sensor nodes failure and traffic overhead increase. Therefore, a VB can be broken and becomes dis-functional. In this paper, we address this problem by proposing a parallel scatter search algorithm for a fault-tolerant VB.

### 2.3. Network Lifetime

The lifetime of a sensor node can be defined as the period from when it starts working to when its energy is depleted [37]. Specifically, the lifetime of any network is the minimum lifetime of all of the sensor nodes in it. Lifetime is one of the most important characteristics of a wireless sensor/ad hoc network since sensor nodes are equipped with energy-limited batteries. Generally, sensor nodes power consumption is resulted from sensors main functions such as sensing, computing, and radio transmission. Among these functional components of sensors, the radio transmission consumes the most portion of the sensor node energy. Therefore, several techniques are proposed to increase network life time by monitoring the power consumption of sensor nodes and taking action before the energy of sensor node/nodes get completely depleted.

A well-known technique is the sleep scheduling, which guarantees the connectivity and coverage simultaneously by switching off some of the unnecessary sensors’ radio and only activating the necessary sensors. Thus, the CDS algorithms can be used to construct multiple virtual backbones to let them work alternatively [38,39]. Wei proposed two CDS construction algorithms, CDS-LL and Extended CDS-LL (E-CDS-LL), to prolong the lifetime of a CDS-based wireless network. The CDS-LL algorithm trades the CDS size for a lifetime by a tunable parameter. The E-CDSLL algorithm balances energy consumption of wireless nodes via dynamically selecting energy-rich nodes with the CDS-LL algorithm to reconstruct the CDS before the CDS is disabled [40].

Wireless energy transfer technology is another promising technique to prolong the lifetime of WSNs [41]. Jin et al. presented an energy replenishment technique for a tree-based WSN by combining wireless information, power transfer and energy harvesting from both radio frequency and ambient energy sources. They analyzed the frame structure and operation from sensing data transmission schedules for all tree-based sensor nodes. Then, they estimated the required energy for each node from the sink node after calculating the available harvested energy. The drawback of their technique include high complexity, careful frame time design according to their proposed principle [42]. The mobile wireless charging vehicle (WCV) has attracted researchers recently to solve the problem within the same context. Tian et al. focused on categorizing sensor nodes according to their importance in the data transmission and uneven energy consumption. They divided sensor nodes into two categories: sensor nodes in ring 0, and sensor nodes in outer ring. They proposed a WCV strategy based on nodes importance and their distance from the base station. In their model, the WCV charges sensor nodes in ring 0 are one by one, and then charges nodes in the outer ring all at once. They also assign a larger battery for each cluster head so that it can charge other sensor nodes within its wireless charging perimeter [43,44].

Employing both VB sleep scheduling and mobile vehicles for wireless charging as a hybrid technique is able to balance the load of WSNs, and provide a reliable energy replenishment for sustainable wireless sensor networks. Specifically, sensor nodes of recently de-scheduled VB can be considered candidate nodes for WCV strategy.

## 3. Parallel Scatter Search Algorithm with Elite and Featured Cores

**Figure 1:**
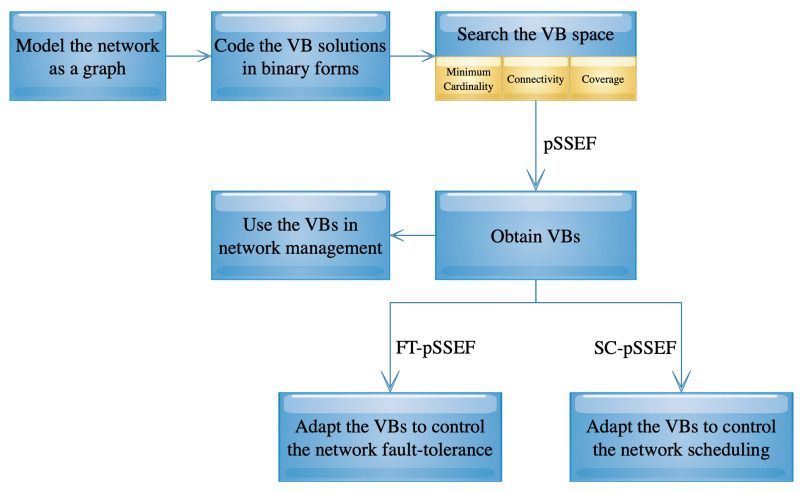
Methodology framework.

The methodology design aims to find VBs of a given network. Then, the obtained VBs can be deployed to manage the network and also to control its fault-tolerance and scheduling in order to extend the network lifetime. Figure 1 shows the framework of the methodology design presented in this section and the next one. First, the network is model as a graph G=(V,E), and a VB can be coded in as 0–1 vector to select the backbone nodes. Then, three main methods are designed to deal with the the considered problem and its applications:

- The parallel Scatter Search with Elite and Featured cores (pSSEF) method: finds optimal or near-optimal VBs,
- the FT-pSSEF method: adapts the obtained VBs to control the network VB fault-tolerance, and
- the SC-pSSEF method: adapts the obtained VBs to control the network VB scheduling.

The design steps and components of the first method are discussed in this section while the rest of the methods are presented in the next section.

In the pSSEF method, the proposed parallelization strategy is based on searching the solution space using different search strategies in parallel. To preserve the elitism and diversity, three types of search are used motivated by elite and featured solutions as well as the ordinary search mechanisms of scatter search. These search mechanisms are run on *p* different cores. The first core is the master one which controls the main search process, and is also concerned with developing the elite solutions. The second core is called featured core and develops connected dominating sets since the connectivity is a main featured of the considered problem. The rest of the (p−2) cores, which are shortly called workers, apply SS procedures on different solution sets simultaneously.

Before explaining the specifics of the proposed method, we begin by defining the representation of the solutions and evaluating their fitness. Then, the scatter search operators are designed including diversification, improvement, reference set update, subset generation and solution combination. Finally, to refine the best obtained solutions, intensification schemes are discussed based on local search and feature refinement.

### 3.1. Solution Representation

The pSSEF method uses a bit vector $X=(x_{1},x_{2},\ldots ,x_{V_{n}}),$ to represent solutions which are node subsets of a given network graph G=(V,E), where $V_{n}$ is the number of vertices or nodes in *G*. The subscript values $1,2,\ldots ,V_{n}$ are related to the corresponding nodes in V(G). If a component $x_{i}$ of *X* has the value 1, then the *i*-th node in V(G) is contained in the nodes subset represented by solution *X*. Otherwise, the solution *X* does not contain this *i*-th node. Therefore, a node subset D⊆V(G) is represented by *X*, where

$$x_{i}=\left\{\begin{array}{cc} 1, & \mathrm{if}\;\mathrm{node}\;i\in D,\\ 0, & \mathrm{if}\;\mathrm{node}\;i\notin D, \end{array}\right.$$

$i=1,2,\ldots ,V_{n}.$

For a given network, the pSSEF method aims to find the best VB which is represented in the above-mentioned binary form. Therefore, the pSSEF method generates different solution candidates to output the best *X* that its corresponding nodes can form a MCDS which works as a VB of the given network.

### 3.2. Fitness Evaluation

As a particular form of objective function, the pSSEF method uses a special fitness function to calculate the solution quality that increases the probability of finding successful solutions with better coverage and connectivity [31,45,46]. Superior guidance of the search using a well-defined fitness function can improve the obtained solutions within fewer iterations, therefore leading to savings in resource expense. Thus, there is a direct relationship between the fitness function and the quality of the obtained solutions. Specifically, the fitness function must distinguish between solutions and give better values for solutions with better features to solve the considered problem. Hence, the pSSEF method invokes a fitness function that measures the solution coverage, connectivity and cardinality as given in the following form:

(1) $$fit\left(X\right)=\alpha \frac{V_{D}}{V_{n}}+\beta \frac{C_{n}}{\sum_{i}^{V_{n}}x_{i}}+\gamma \frac{V_{n}-\sum_{i}^{V_{n}}x_{i}}{V_{n}},$$

where $V_{D}$ is the number of nodes dominated by the subset of nodes *D* represented by the solution *X*, $C_{n}$ is the number of nodes in largest connected set in *D*, and α, β and γ are weight parameters with α+β+γ=1.

The fitness function represented by Equation (1) can be considered to be a weighted sum of multi-objectives. To be more specific, it consists of three objectives as given below.

- The first objective is the extent to which the solution covers the graph, which represented by the term
  $$\frac{V_{D}}{V_{n}}.$$
- The second objective is to measure the solution connectivity which is given by the second term
  $$\frac{C_{n}}{\sum_{i}^{V_{n}}x_{i}}.$$
- The third objective is the relative number of nodes in the solution which can be measured by the term
  $$\frac{V_{n}-\sum_{i}^{V_{n}}x_{i}}{V_{n}}.$$

It can be seen that the first objective (coverage) and the second objective (connectivity) are to be maximized. The third objective is related to the solution cardinality which should be minimized. Therefore, the third objective is formulated so that its maximization is equivalent to solution cardinality minimization. Then, we can say that the considered MCDS problem can be reformulated in the following form:

(2) $$\max_{X\in B}fit\left(X\right),$$

where $B={\left\{0,1\right\}}^{V_{n}}$, and fit(X) is defined by Equation (1).

In [47,48], the fitness function represented by Equation (1) was firstly developed to contain two objectives; coverage and cardinality, since the main problem was to find minimum dominating sets. Then, the function form is modified to contain the additive term of connectivity in [30,31,46] in order to deal with finding minimum connected dominating sets. We use that last modification of the fitness function in the pSSEF method as shown in Equation (1).

### 3.3. Scatter Search Procedures

The pSSEF method uses common search mechanisms in SS in addition to some intensification and parallel mechanisms. The details of the SS procedures are explained as follows.

#### 3.3.1. Diversification Generation

The pSSEF method applies the diversification generation procedure to build a large population set *P* of diverse solutions [7,49]. Therefore, a large enough number $P_{size}$ of solutions are generated randomly over the solution search space ${\left\{0,1\right\}}^{V_{n}}.$

#### 3.3.2. Improvement

The improvement procedure transforms a trial solution into another one with higher quality. If there is no improvement in the input trial solution results, then the enhanced solution is considered to be the same as the input solution [50]. In pSSEF method, the invoked improvement procedure is called local search and will be explained in the intensification procedures later. The local search procedure tries to improve the solution features of coverage and cardinality.

#### 3.3.3. Reference Set Update

The Reference Set (RefSet) is the iterate solution set in SS similar to the population in Evolutionary Algorithms (EAs). Unlike other EAs, the RefSet has limited number of solutions compared to populations in EAs. The reference set update procedure is applied to initialize the RefSet in the search beginning and also to update it in every generation. To generate an initial RefSet, collection of both high quality solutions and diverse solutions are selected from *P*. Specifically, the RefSet with size $Ref_{Size}$ consists of two different subsets:

- RefSet${}_{1}$ that contains *b* elite solutions, and
- RefSet${}_{2}$ that contains $\left(Ref_{Size}-b\right)$ diverse solutions.

The reference set update procedure copies the *b* best solutions from *P* to RefSet${}_{1}$ and deletes them from *P*. For each solution in the updated *P*, the minimum of distances to solutions in RefSet${}_{1}$ is computed. Then, the $\left(Ref_{Size}-b\right)$ solutions with the maximum values of these minimum distances are selected and added to RefSet${}_{2}$ [51].

At the end of each generation, the RefSet is updated using the same procedure mentioned in creating the initial RefSet, but the set of all solutions generated in this generation are used instead of P.

#### 3.3.4. Subset Generation

The pSSEF method operates on the RefSet to produce new solutions by combining each pair of solutions in it. Specifically, the subset generation procedure relies on generating all possible pairs from the set of solutions in the RefSet. This results in $\left[Ref_{Size}\left(Ref_{Size}-1\right)/2\right]$ subsets of size 2.

#### 3.3.5. Solution Combination

The solution combination procedure uses the generated solution subsets to produce new solutions by applying the three-point cut method. In each solution subset, three cut points are selected randomly and the bits between the three points are swapped between the solutions to create new solutions [50].

### 3.4. Intensification Procedures

Elite solutions obtained during the search process are enhanced using two procedures to achieve a faster and a better performance. The details of these procedures are explained as the following.

#### 3.4.1. Local Search

**Figure 2:**
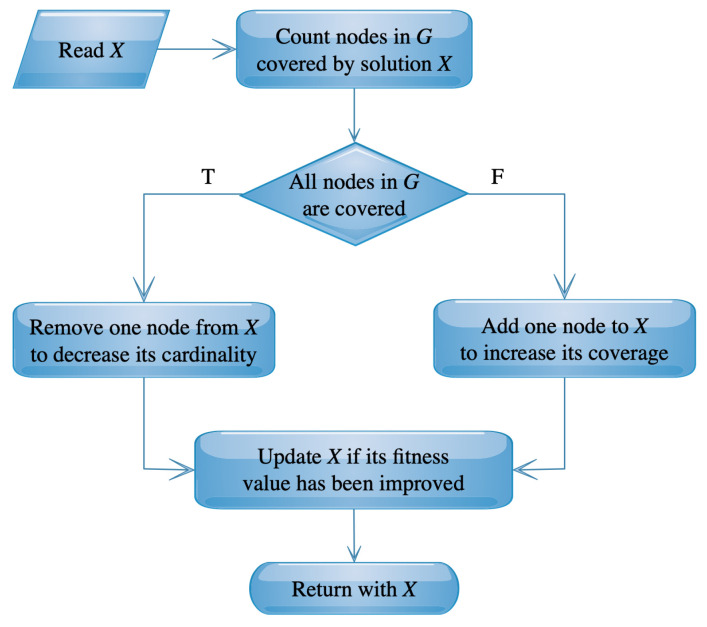
Main steps of local search.

The pSSEF method applies the local search (LS) mechanism in order to improve the fitness of the obtained solutions by adding or deleting some nodes in these solutions. The addition or removal of nodes is depending on the domination feature of solution *X*. If *X* is a DS, then the LS procedure tries to improve its cardinality by deleting some nodes. On the other hand, if *X* is not a dominating set, then the LS procedure tries to improve it by adding some nodes to hopefully get a new dominant solution. The main processes of the LS procedure are illustrated in Figure 2. The addition or removal of nodes is directly proportional or inversely proportional, respectively, with degrees of nodes contained in *X*. This means that the node adding targets the nodes with higher degrees, while the node deleting targets the others that have lower degrees. This process is repeated $n_{step}$ times.

#### 3.4.2. Final Intensification

In order to find a MCDS, the generated connected dominating solutions are saved in a set denoted by the CD set. The final intensification procedure attempts to improve the cardinality of these solutions by removing all unnecessary nodes without losing the solution coverage or connectivity.

### 3.5. Parallel Procedures

The proposed method is operated on *p* different cores, and the search procedures are applied on these cores in parallel. Two of these core are distinctive. The first one is called “Elite and Master Core” which is the main core that controls the main search process, and it is also responsible for producing new elite solutions. The second core is called “Featured Core” which develops new connected solutions. The rest of cores are called “workers” which generate general solutions represented different possible VBs and pass these solution of the main cores.

#### 3.5.1. Elite and Featured Cores

The Elite and Master Core applies the search procedures on special RefSet called Elite RefSet which contains the $Ref_{Size}$ best solutions obtained so far. The Featured Core uses another distinctive RefSet called Featured RefSet which contains the best connected solutions obtained so far. These best connected solutions are named featured solutions.

#### 3.5.2. Migration

Within a certain number of generations, called the migration interval and denoted by *m*, the previously mentioned search procedures are applied periodically on each RefSet without migrating any individuals into this RefSet. When the migration interval is achieved, the migration process is initiated to add new elite and featured solutions into each worker RefSet. Moreover, the Elite and Master Core is responsible for collecting all elite and featured solutions from all cores, ordering them and sending the best of them to other cores.

### 3.6. The pSSEF Algorithm

The pSSEF algorithm starts with setting the initial parameters at the Elite and Master Core and broadcasts the input data to all other cores. In general, parallelism has introduced new degrees of freedom to algorithm design approaches. For investigating the effectiveness of using parallel systems, two versions of the pSSEF algorithm are proposed. The main difference between them is depending on:

- How each worker selects and updates its reference set.
- How the migration process is applied.

**Figure 3:**
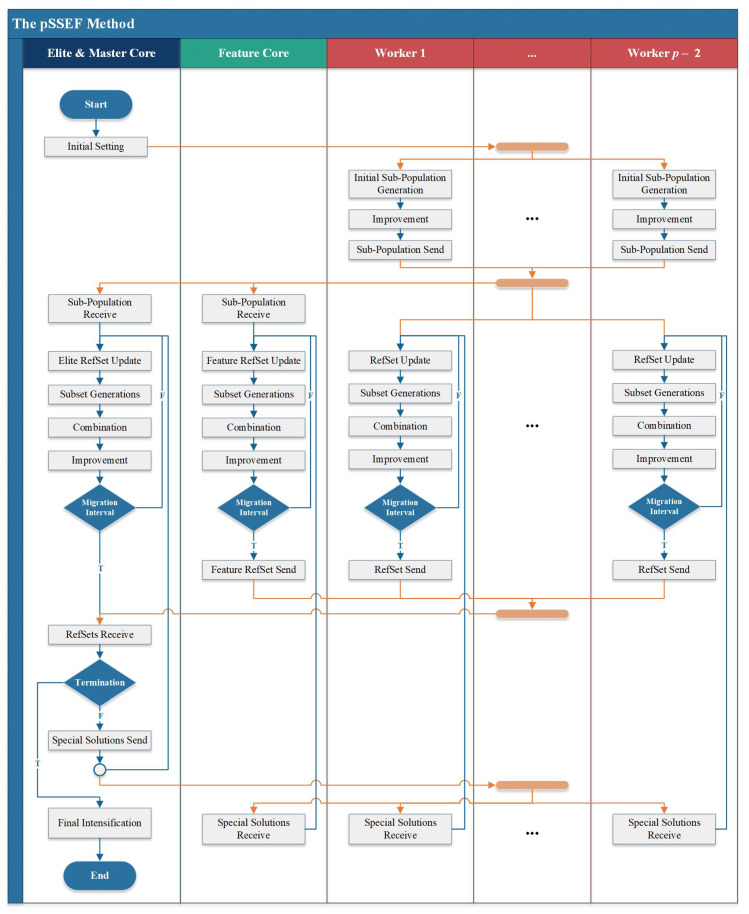
The pSSEF flowchart.

The pSSEF versions; pSSEF1 and pSSEF2, follow the main structure represented in Figure 3. The details of these versions are explained in the following sub-sections.

#### 3.6.1. The First Version pSSEF1

In the pSSEF1 algorithm, each worker starts to generate its own initial sub-population of size $P_{size}$ and improves the sub-population members using the LS procedure. Then, all workers send their sub-population to the Elite and Master and Featured Cores. The Elite and Master Core selects the best $P_{size}$ solutions of higher quality among all generated sub-populations. The Featured Core selects its sub-population from the best connected dominating solutions among all generated sub-populations. Then, each core starts the main loop including four main steps; RefSet update, subset generation, solution combination and improvement. The loop of these scatter search procedures is repeated over *m* consequent generations, where *m* is the size of the migration interval. After that, all obtained RefSet are collected in the Elite and Master Core which in turn orders all solutions in the received RefSets and sends some of distinctive solutions to them to all workers. Specifically, the Elite and Master Core selects the (p−2) best solutions and the (p−2) best featured (connected) solutions. Then, one solution from these best solutions and one solution from these featured solutions are randomly sent to each worker. Moreover, the Elite and Master Core sends the best $Ref_{size}$ to the Featured Core. Then, a new migration interval is applied unless the termination criteria are met. Before terminating the algorithm, the final intensification procedure is applied to improve the best obtained solutions.

#### 3.6.2. The Second Version pSSEF2

The pSSEF2 algorithm works the same as the first version, except for three main operations as follows.

- The initial Featured RefSet is generating by selecting 20% of its members from the best connected dominating solutions among all workers sub-populations. Then, the Featured Core computes the minimum hamming distances between each of the rest of unselected solutions and the selected connected dominating ones. Solutions with the largest distances are chosen to complement the remaining 80% of the Featured RefSet members.
- At the beginning of each migration interval, the Featured RefSet is updated in the same way that it was initialized.
- At the end of each migration interval, a refinement process is applied by calling the final intensification procedure to improve the obtained solutions in the CD solution set.

## 4. Algorithmic Implementations to Network Virtual Backbone Fault-Tolerance and Scheduling

**Figure 4:**
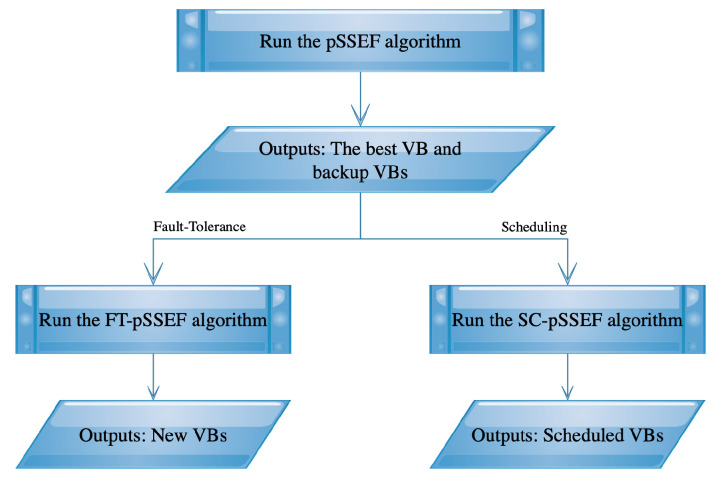
Implementations of the pSSEF algorithm to the VB fault-tolerance and scheduling.

In this section, two extensions of the pSSEF algorithm are proposed to deal with the fault-tolerance and scheduling problems in network virtual backbones. After running the pSSEF algorithm, we can get the best VB and some other alternatives of VBs denoted as backup VBs. Then, two additive algorithms use these obtained VBs to control the network fault-tolerance and scheduling as shown in Figure 4.

### 4.1. Virtual Backbone Fault-Tolerance

A modified version of the pSSEF algorithm is composed to deal with the network virtual backbone fault-tolerant. This modified version is denoted by the FT-pSSEF method and aims to tackle the failure that may occur in the VB. The FT-pSSEF algorithm uses two additive mechanisms; solution reconnecting and solution coverage, which are explained before presenting the FT-pSSEF algorithm formally.

#### 4.1.1. Solution Reconnecting

The solution reconnecting mechanism aims to repair the disconnection between the VB nodes caused by the failure of any node. Specifically, the Solution Reconnecting procedure tries to add a minimal number of nodes to the best solution $X_{best}$ if it is not connected. First, the Solution Reconnecting procedure finds the largest maximal connected component *C* in $X_{best}$. Then, all other remaining nodes in the $X_{best}$ are collected in a set denoted by $\bar{C}$. Then, the Solution Reconnecting procedure selects two nodes $n_{1}\in C$ and $n_{2}\in \bar{C}$ randomly. The selection probability of these two nodes are proportional to their degree. Then, all intermediate nodes along the shortest path from $n_{1}$ to $n_{2}$ are added to $X_{best}$. This node addition process is repeated until solution $X_{best}$ is connected. The formal description of the Solution Reconnecting procedure is stated in Algorithm 1.
**Algorithm 1** Solution Reconnecting.Set *C* equal to the nodes in the largest maximal connected component in $X_{best}$, and set $\bar{C}$ equal to set of other nodes contained in the in $X_{best}$.If $|\bar{C}|=0$, return.Randomly select two nodes, $n_{1}\in C$ and $n_{2}\in \bar{C}$. The selection probability of these nodes is proportional to their degree.Find the shortest path from $n_{1}$ to $n_{2}$.Set η to be the set of all intermediate nodes in the obtained shortest path.Update $X_{best}$ to contain all new nodes in η.If $X_{best}$ is connected, then return. Otherwise, go to Step 1.

#### 4.1.2. Solution Coverage

The Solution Coverage procedure is another mechanism used by the FT-pSSEF algorithm to refine the coverage of the best solution $X_{best}$. It is assumed that solution $X_{best}$ represents a connected set of nodes. The Solution Coverage procedure refines $X_{best}$ by adding some nodes to it to increase its coverage if it is not a dominating set. This addition is randomly and proportional to the node degree, which means the nodes with a high degree have a high probability to be added to $X_{best}$. Therefore, this procedure tries to increase the coverage of the solution without losing its connectivity property. The formal description of the Solution Coverage mechanism is stated in the following Algorithm 2.
**Algorithm 2** Solution Coverage.Collect the nodes represented by $X_{best}$ in set ξ and the other nodes in set $\bar{\xi }$.If ξ is a dominating set, return.Set $X_{trial}=X_{best}$.Randomly select a node from $\bar{\xi }$ which is adjacent to a node in ξ. The selection probability of a node is proportional to its degree.Set the corresponding component in $X_{best}$ to the selected node equal to 1.If $fit\left(X_{trial}\right)>fit\left(X_{best}\right)$, then set $X_{best}=X_{trial},$ update the node sets ξ and $\bar{\xi }$, and go to Step 2.

#### 4.1.3. The FT-pSSEF Algorithm

**Figure 5:**
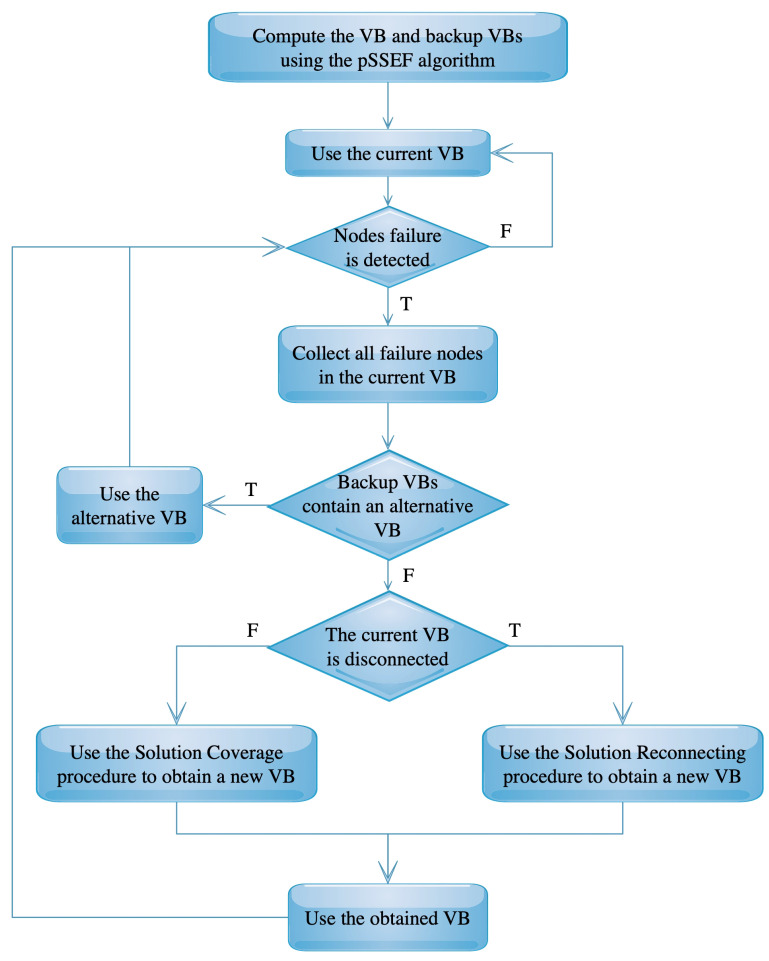
The FT-pSSEF flowchart.

Since the virtual backbone nodes are prone to failure, a set of different solutions can be obtained from the pSSEF algorithm. These backup solutions are already saved in the CD set. The FT-pSSEF algorithm uses these solutions for providing fault-tolerance to the given network in two ways. First, the FT-pSSEF algorithm searching for another VB from the backup solutions provided by the main pSSEF algorithm. The selected backup solution should not contain any failed node. Second, if there is no such backup solution that matches the specifications in the first step, the FT-pSSEF algorithm repairing the broken VB. The repair process can be achieved by increasing the connectivity between its nodes using the Solution Reconnecting procedure, or by improving the node coverage in the virtual backbone using the Solution Coverage procedure. Figure 5 shows the main structure of the FT-pSSEF algorithm.

### 4.2. Virtual Backbone Scheduling

**Figure 6:**
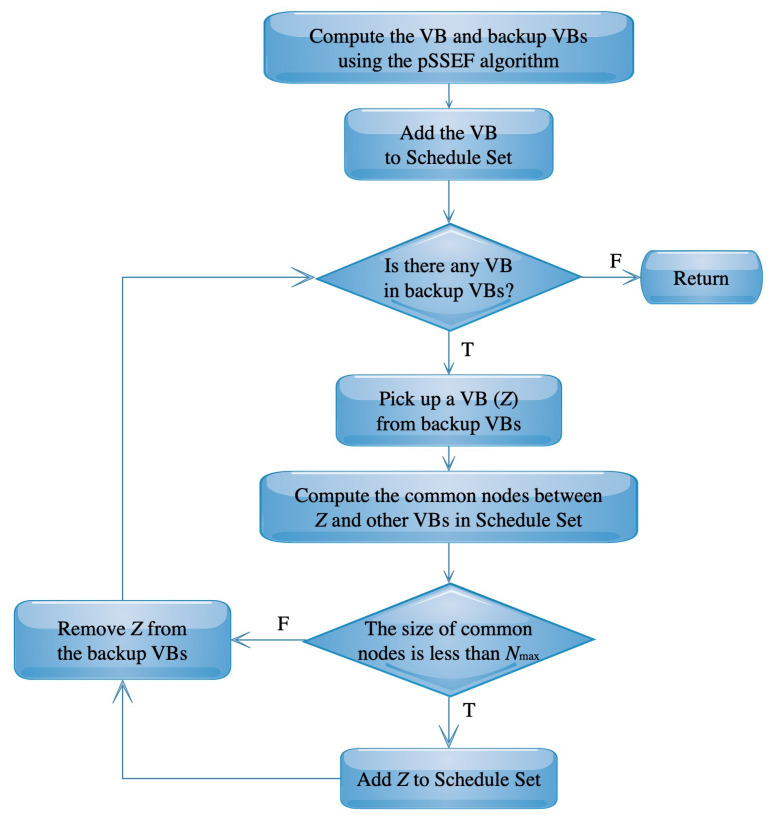
The SC-pSSEF flowchart.

Another modified version of the pSSEF algorithm is designed to deal with the virtual backbone scheduling, which is called the SC-pSSEF algorithm. This modified algorithm aims to find multiple VBs using the best solutions for MCDS provided by the pSSEF algorithm which are saved in the CD set. During the search process, the SC-pSSEF algorithm finds and stores different connected dominating sets with maximum diversity and minimum cardinality. The SC-pSSEF uses the best VB and backup VBs obtained by the original steps of the pSSEF algorithm, then the SC-pSSEF distinguishes between the obtained VBs to find better virtual backbone scheduling. Figure 6 shows how the SC-pSSEF works to filter the obtained VBs and saves the suitable ones in a set called Schedule Set. The degrees of symmetry between the VBs in this set are limited, as the common nodes in each VB pair do not exceed a pre-defined number $N_{\max}$.

**Figure 7:**
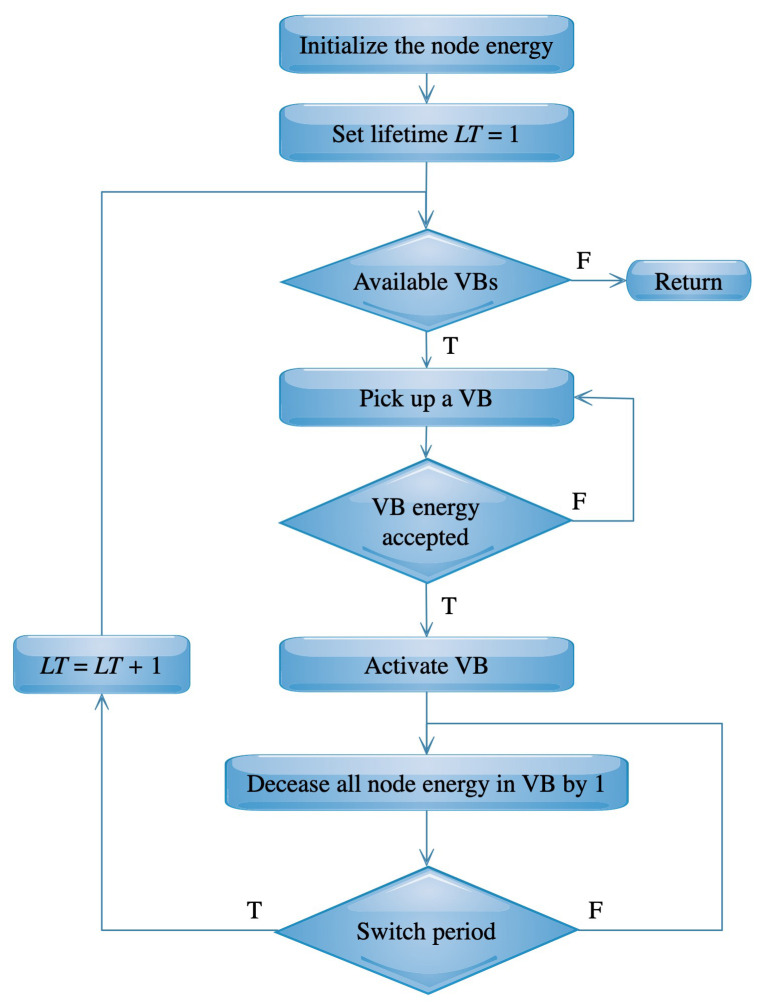
Step for computing the network lifetime LT in rounds.

In order to measure the efficiency of a VB scheduling process, the network lifetime can be estimated using the following mechanism. First, we define the node lifetime as the time period from when it begins to function to when its energy is exhausted. Moreover, the VB lifetime is the minimum life of all its nodes [10]. A schedule can be define as a set of VBs that are working sequentially in order to increase the network lifetime. Formally, we need to find a set of VBs; $VBS=\{VB_{1},\ldots ,VB_{\mu }\},$ and each backbone $VB_{i}$ works for time $T_{i}$, where i=1,…,μ. Then, the schedule is represented by a set of tuples $\left\{\left(VB_{1},T_{1}\right),\ldots ,\left(VB_{\mu },T_{\mu }\right)\right\}$. Since the backbones rotate after each period of time called round (τ), and the network lifetime (LT) is counted in rounds as shown in Figure 7. We assume that each node has an initial energy (En) which is decreased by 1 after each time its VB is used. A VB is available whenever all of its nodes has an energy greater than 0. The available VBs are exchanged each round, i.e. the current VB is switched off after running τ times and the next VB is switched on after that. The lifetime computing algorithm starts with $VB_{1}$, after τ times it switches to $VB_{2}$, and so on. After the algorithm reaches $VB_{\mu }$, it switches again to $VB_{1}$. The full steps of the life time algorithm is illustrated in Figure 7.

## 5. Numerical Results

The pSSEF algorithm and its extensions was programmed using MATLAB and run on Dell Precision Workstation 7610 with dual Xeon E5-2663-v3 2.8GHz processors including 20 cores. In this experimental section, we analyze the effectiveness of the pSSEF method and its extensions on a group of network graphs as well as their application in virtual backbone fault-tolerance and scheduling problems. First, we discuss the main test bed and parameters setting. Then, we investigate the performance of the proposed algorithm and find its best implementation settings. Moreover, the results of the proposed methods are compared against the results of other benchmark methods presented in [27,45,46]. To measure the performance of the pSSEF method, comparisons are made using different quantities:

- Minimum approximate domination number (Min) which is the number of nodes in the best connected dominating solution found in all independent runs.
- Average approximate domination number (Avg) which is the average number of nodes in the best connected dominating solution found in each independent runs.
- Processing time in seconds which is the average of the code running time over independent runs.

These performance metrics are used by the compared methods and are commonly used in the literature. Finally, the results of the FT-pSSEF and SC-pSSEF methods on the virtual backbone fault-tolerance and scheduling problems, respectively, are presented and discussed.

### 5.1. Test Bed and Parameter Setting

A large set of sensor/ad hoc network clustering instances are generated. In which, eight different networks (instances) occupy the same area with different number of nodes or vertices. The network sizes very from 80 nodes up to 400 nodes as given in Table 1. In all networks, each node is connected to its adjacent nodes if the distance between them is not more than a transmission range *R*. The experiments were performed 20 times for each of the considered networks with different transmission ranges.

The setting of the pSSEF, FT-pSSEF and SC-pSSEF parameters are shown in Table 2 with their assigned values. These chosen values are based on our numerical experiments or according to known common settings in the literature [45,46,52].

### 5.2. Performance Analysis

The performance of the main proposed method is analyzed through several experiments using different prospective. First, the performance between the two versions; pSSEF1, and pSSEF2 is compared in order to choose the best setting of the main method. Then, another comparison is done between the pSSEF method and the the sequential version ASSA-MCDS [45]. Actually, the pSSEF method extends and modifies our previous developed sequential scatter search ASSA-MCDS into parallel environment. In this comparison, we take into account another comparing quantity which is the computational cost in processing time. Finally, the performance of the pSSEF code is analyzed using different size of computing cores.

#### 5.2.1. Performance Comparison between the pSSEF1 and pSSEF2 Versions

**Figure 8:**
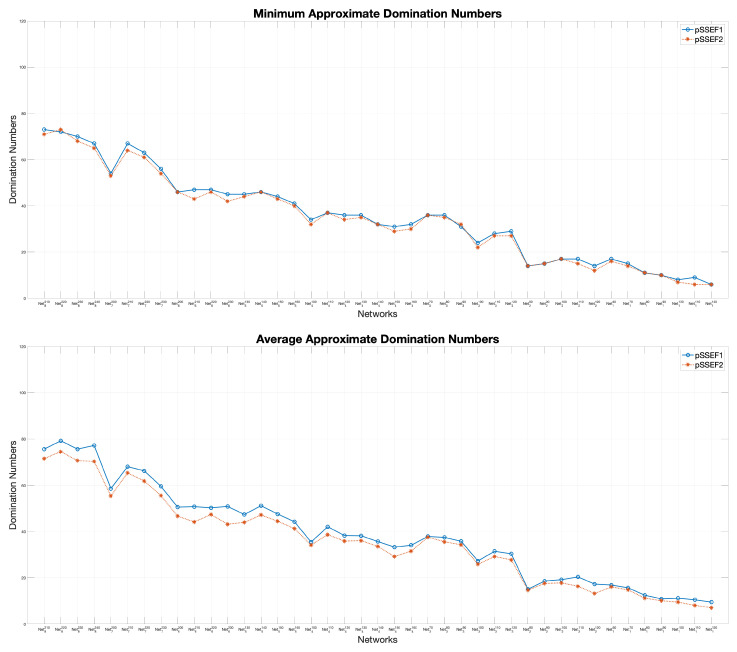
Performance comparisons between pSSEF1 and pSSEF2.

The performance comparisons between the two versions; pSSEF1, and pSSEF2, are demonstrated in Figure 8. Both algorithms have been run 20 times using 4 cores and their parameter values are set as in Table 2. The obtained experimental results show that pSSEF2 noticeably outperform pSSEF1 for all network instances in terms of the average and domination number. Therefore, in all of the following results and discussion the main proposed pSSEF algorithm follows the settings of the pSSEF2 algorithm.

#### 5.2.2. Performance Comparison between Sequential and Parallel Scatter Search

**Figure 9:**
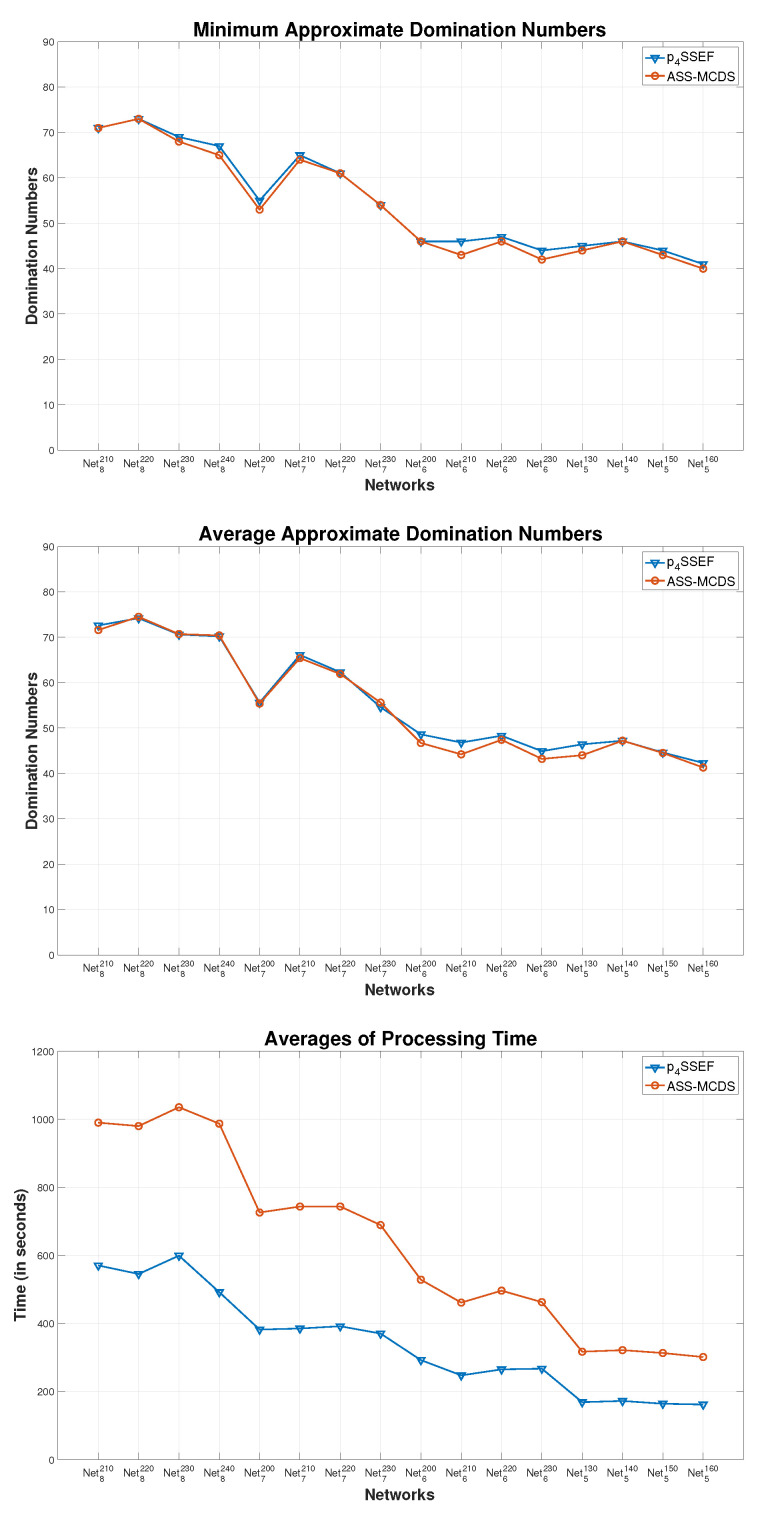
Metric comparisons between pSSEF and ASS-MCDS on big-size networks.

In this comparison, we compare pSSEF, which is a parallel modified version of the sequential method ASSA-MCDS [45]. The results of this comparison are reported in Figure 9. In this figure three comparison metrics are considered; the average, minimum approximate domination numbers and the computational cost. Both methods have the same number of runs for each graph, which is 20 times. The comparison between the two methods shows that pSSEF noticeably outperforms ASSA-MCDS for all network instances described. Moreover, the pSSEF method achieved superior performance against the ASSA-MCDS method for all instances in terms of the computational cost where the processing time is reduced by almost 50%. This is due to the parallel design of the pSSEF method.

**Figure 10:**
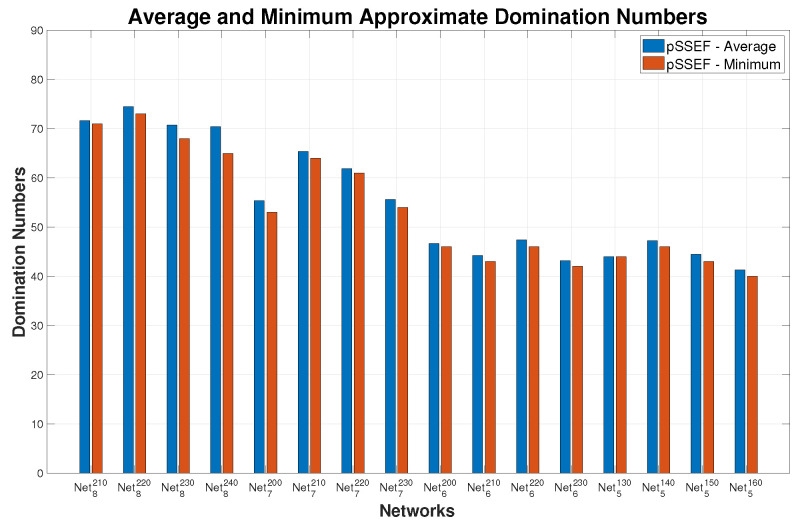
Comparisons between the average and minimum connected domination number of pSSEF.

Another significant indication for the performance evaluation of the proposed algorithm is the convergence between the results of the average and the minimum solutions that acquired from 20 independent runs on the network instances. The comparison results in Figure 10 show that the average achieves a considerable converge to the minimum domination number for all network instances. Thus, the pSSEF method is more stable than the sequential method ASSA-MCDS.

#### 5.2.3. Parallel Performance of the pSSEF Method

The parallel performance of the pSSEF method is tested on different number of cores. Specifically, the five parallel versions of the proposed method are tested, each version uses one Master and Elite core, one Diverse core and different numbers of Worker cores as follows:

- p${}_{4}$SSEF: 2 Worker cores.
- p${}_{8}$SSEF: 6 Worker cores.
- p${}_{12}$SSEF: 10 Worker cores.
- p${}_{16}$SSEF: 14 Worker cores.
- p${}_{20}$SSEF: 18 Worker cores.

**Figure 11:**
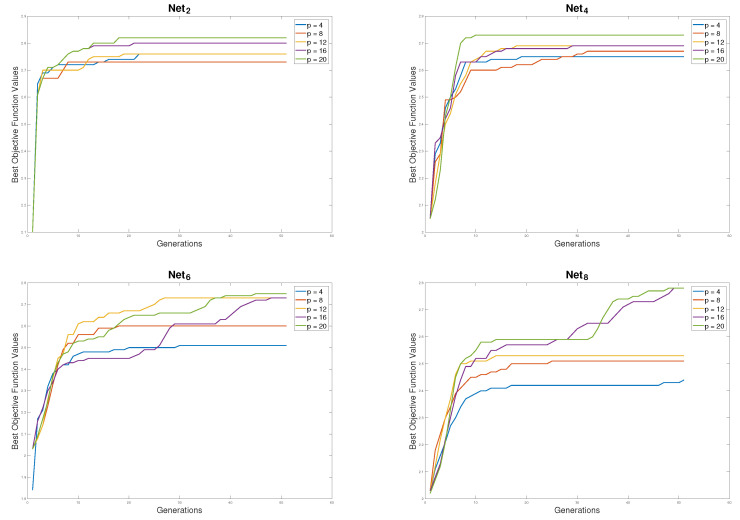
Parallel performance of the pSSEF with $Net_{2}80$, $Net_{4}100$, $Net_{6}200$ and $Net_{8}210$.

The results of running these parallel versions of the proposed method are shown in Table 3 and Figure 11. The minimum and average approximated domination numbers of the obtained MCDS are shown in Table 3 for the 8 test networks. The results of this table show that the performance of the proposed method become better when the number of cores is increased since the average domination numbers are decreased. Figure 11 also shows the same better performance since the fitness values are increased when number of cores is increased.

**Figure 12:**
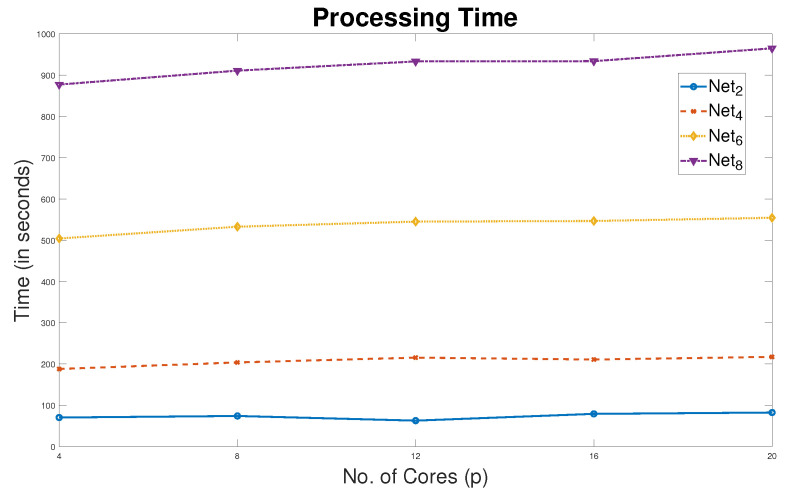
Parallel processing time of pSSEF with $Net_{2}^{80}$, $Net_{4}^{100}$, $Net_{6}^{200}$ and $Net_{8}^{210}$.

Processing times for four networks over different number of cores are shown in Figure 12. For each network, the processing time is almost unchanged which means there is no much improvement in speed-up. This is because each worker applies the search operations on the same size RefSet, regardless of the number of cores used. This means that processing data is not divided over cores. In fact, comparing with the sequential method, a significant improvement in processing time is observed. However, the improvement in solution qualities is not as good as the improvement in the processing time. This has motivated us to implement the pSSEF algorithm to improve solution qualities in stead of improving the time processing time. It is worthwhile to mention that in all of the following discussion we use the p${}_{4}$SSEF results which is simply denoted by the pSSEF.

### 5.3. Results and Discussion

In this section, the results of the proposed methods are discussed compared to other benchmark methods. Moreover, we discuss the application results on the virtual network fault-tolerance and scheduling problems.

#### 5.3.1. Performance Comparison of the pSSEF and Other Benchmark Methods

The results of the pSSEF method are compared with those of the following methods:

- GRASP: A greedy randomized adaptive search procedure for connected dominating set problems [27].
- MA-MCDS: Memetic algorithm for minimum connected dominating set [46].
- SA-MCDS: Simulated annealing for minimum connected dominating set [46].
- ASSA-MCDS: Adaptive scatter search algorithm for the minimum connected dominating set [45].

**Figure 13:**
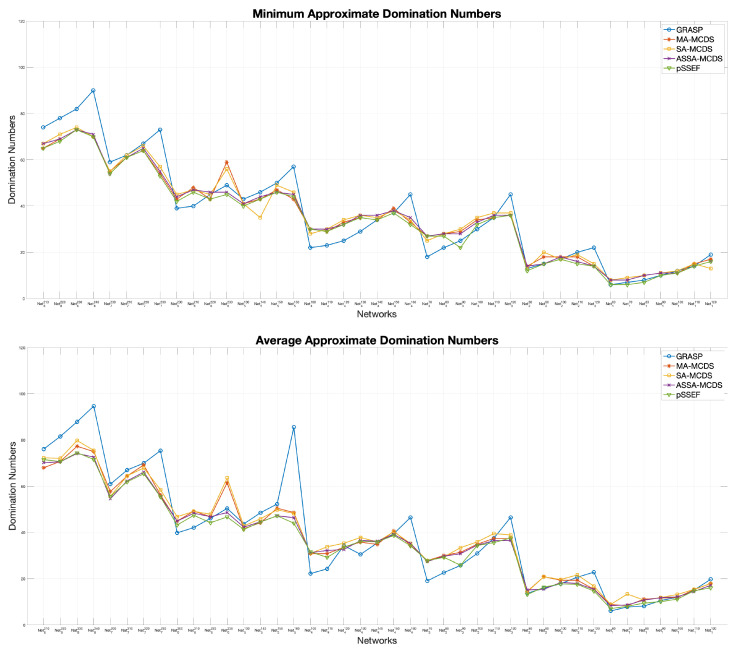
Compared results for big-size networks.

Table 4 displays the minimum number (Min) and average values (Avg) results for all instances in Table 1 obtained by applying the pSSEF method and the other methods mentioned above. The best of the minimum and average approximate domination numbers of the acquired solutions are highlighted in boldface for each row in the table. By examining the results shown in Table 4 precisely, we can generally conclude that our proposed method gives the best result for most network instances, particularly those with a large number of wireless nodes. Moreover, the proposed method gives the best overall average and could beat the compared methods in most of the used networks as shown at the bottom of Table 4. The disparity in performance between different methods on all networks can be clearly shown in the Figure 13. It worthwhile to notice the performance of VB sizes with high density networks, it is reasonable that the sizes of the VBs decrease with increasing of transmission ranges. However, we note that in a few networks with smaller transmission ranges such as $Net_{5}^{130}$, $Net_{6}^{200}$, $Net_{7}^{200}$ and $Net_{8}^{210}$, the sizes of the obtained VBs is less than their values with larger ranges as in $Net_{5}^{140}$, $Net_{6}^{210}$, $Net_{7}^{210}$ and $Net_{8}^{220}$, respectively. This is because of the connectivity recommendations [53,54], which is to increase the nodes degrees to ensure that the generated graphs with smaller transmission ranges are connected.

#### 5.3.2. Virtual Backbone Fault-Tolerance Results

**Figure 14:**
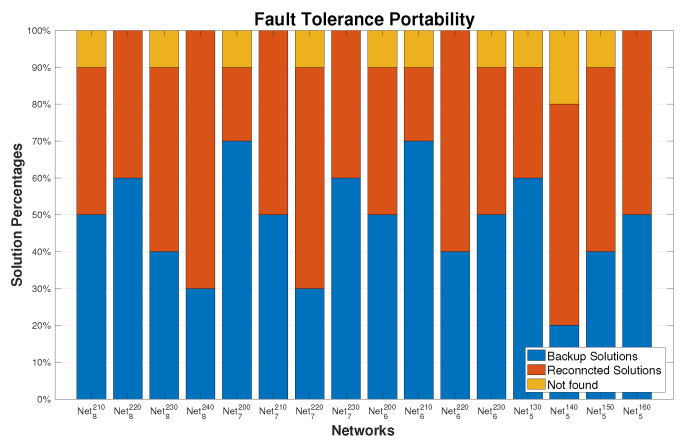
Determining the fault-tolerance Backbone on big-size networks.

The performance of the proposed algorithm FT-pSSEF for the fault tolerant virtual backbone in sensor/ad hoc clustering networks shown in Figure 14, for each network the FT-pSSEF was run 20 times with different failure node. The results in Figure 14 show promising efficiency of the FT-pSSEF method on recovering faults that occur in the network instances. More precisely, around 48.125% of the faults have been recovered by the backup solutions, 45% have been recovered by reconnecting the original solution and the rest of the faults which is around 6.875% have not been covered. In such cases, the pSSEF algorithm is applied again to acquire a new solution.

#### 5.3.3. Virtual Backbone Scheduling Results

**Figure 15:**
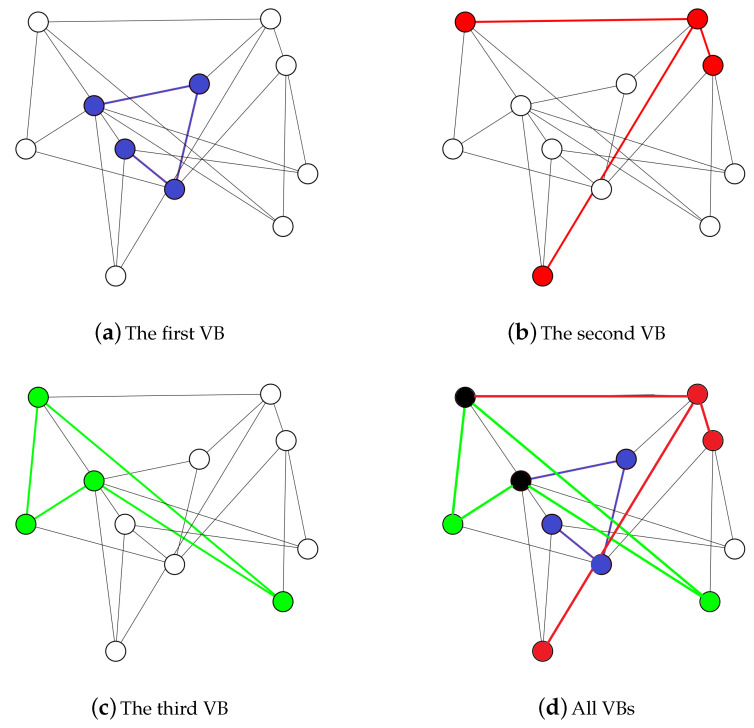
N1 with three virtual backbones.

**Figure 16:**
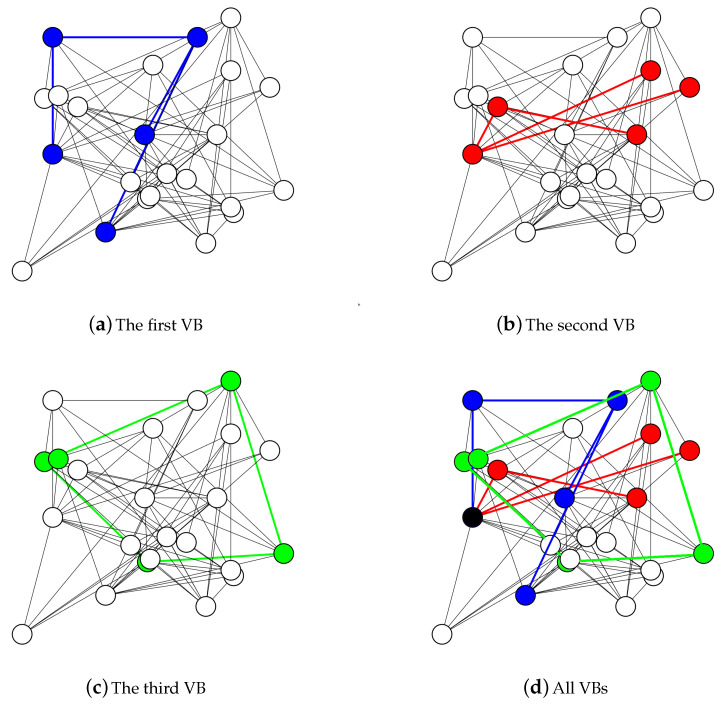
N2 with three virtual backbones.

**Figure 17:**
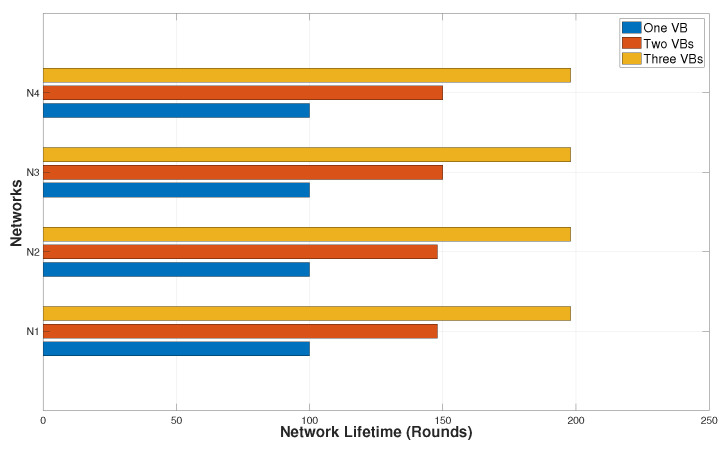
Network lifetime.

The performance of the proposed algorithm SC-pSSEF to determine the a set of VBs for scheduling is tested using four test networks N1, N2, N3 and N4, with node numbers equal to 11, 23, 47 and 95, respectively. The nodes are randomly deployed to a fixed area of 100 × 100. Figure 15 and Figure 16 shows instances of multiple backbones found by the SC-pSSEF algorithm for N1 and N2, respectively. By assuming that backbone nodes consume 1 unit of energy per unit of time, Figure 17 illustrates the benefit of using these multiple Backbones. In this figure, the network lifetime method is called to compute the lifetime of the considered networks with different number of VBs. The comparisons in Figure 17 are between the network lifetimes using 1, 2 or 3 VBs. As showing in the figure, two backbones can prolong the network lifetime by almost 50% whereas three can prolong the network lifetime by almost 98%.

## 6. Conclusions

In this paper, we investigated the problem of constructing a virtual backbone network for controlling either wireless ad hoc networks or wireless sensor networks. In graph theory, this problem is equivalent to finding the minimum connected dominating sets. We proposed the pSSEF method that based on the parallelization technique applied on a multi-core system. A special core called the elite have been introduced to maintain the elitism. Another core called featured core been introduced to maintain the feature of special connected solutions. The rest cores are designed to maintain search for different solution types according to scatter search and new designed search procedures. Also two versions, fault-tolerance pSSEF (FT-pSSEF) and scheduling pSSEF (SC-pSSEF), are proposed to deal with the virtual backbone fault-tolerance and scheduling problems.

The results of the pSSEF method are compared with other methods in the literature using standard benchmarks. The results proved that the pSSEF method could obtain better solutions. Moreover, the results indicated that the pSSEF method is more efficient than the sequential method, especially in the processing time where the parallel algorithm is approximately twice faster than the sequential one. A parallel implementation using several numbers of cores is also designed to improve the solution qualities. The applications of the proposed method in virtual backbone fault-tolerance and scheduling demonstrate its ability to tolerate node failures and prolong the network lifetime, respectively.

## Figures and Tables

**Table 1:** Networks used in testing.

Network	No. of Vertices $\left(n_{V}\right)$	Area (L×L)	Range(R)
$Net_{1}$	80	400×400	60–120
$Net_{2}$	100	600×600	80–120
$Net_{3}$	200	700×700	70–120
$Net_{4}$	200	1000×1000	100–160
$Net_{5}$	250	1500×1500	130–160
$Net_{6}$	300	2000×2000	200–230
$Net_{7}$	350	2500×2500	200–230
$Net_{8}$	400	3000×3000	210–240

**Table 2:** Parameter setting.

Parameter	Definition	Value
$P_{size}$	Population size	150
$n_{step}$	Number of local search iterations	10
*m*	Migration interval	10
$Ref_{size}$	Size of RefSet	15
CD${}_{size}$	Size of the CD set	50
$N_{\max}$	Max no. of common nodes in VB scheduling	γ(G)/4
Eny	The energy of the node in units	100
τ	The number of iterations for VB switching	1

**Table 3:** Comparison results for network clustering.

Networks		p${}_{4}$SSEF-MCDS		p${}_{8}$SSEF-MCDS		p${}_{12}$SSEF-MCDS		p${}_{16}$SSEF-MCDS		p${}_{20}$SSEF-MCDS	
ID	*R*	Avg	Min	Avg	Min	Avg	Min	Avg	Min	Avg	Min
$Net_{1}$	60	16.14	16	16.12	16	16.08	16	16.02	16	16.02	16
$Net_{2}$	80	14.74	14	14.54	14	14.60	14	14.36	14	14.28	14
$Net_{3}$	70	37.64	36	37.42	36	36.80	36	36.92	36	36.56	36
$Net_{4}$	100	34.20	32	34.28	32	33.98	32	34.04	32	33.74	32
$Net_{5}$	130	44.04	44	44.06	44	44.04	44	44.02	44	44.02	44
$Net_{6}$	200	46.72	45	46.06	45	46.20	45	45.96	45	45.80	45
$Net_{7}$	200	55.44	53	55.00	53	54.28	53	54.40	53	54.04	53
$Net_{8}$	210	71.62	70	71.40	70	71.44	70	71.08	70	70.68	70

**Table 4:** Comparison results for network clustering.

Networks		GRASP		MA-MCDS		SA-MCDS		ASSA-MCDS		pSSEF	
ID	*R*	Avg	Min	Avg	Min	Avg	Min	Avg	Min	Avg	Min
$Net_{1}$	60	19.8	19	18.0	17	18.0	**13**	17.2	16	16.1	16
$Net_{1}$	70	15.1	**14**	15.3	15	15.4	15	14.5	**14**	14.8	**14**
$Net_{1}$	80	12.0	12	11.9	**11**	13.2	12	12.2	**11**	11.2	**11**
$Net_{1}$	90	10.6	**10**	11.7	11	11.9	11	11.8	11	10.1	**10**
$Net_{1}$	100	8.2	8	11.2	10	10.8	10	10.8	10	9.5	**7**
$Net_{1}$	110	7.8	7	8.4	8	13.4	9	8.7	8	8.1	**6**
$Net_{1}$	120	6.1	**6**	8.9	8	8.9	8	8.4	8	7.1	**6**
$Net_{2}$	80	22.9	22	15.5	**14**	16.8	15	15.4	**14**	14.7	**14**
$Net_{2}$	90	20.7	20	19.4	18	21.7	19	18.0	16	17.6	**15**
$Net_{2}$	100	17.9	**17**	19.4	18	19.7	**17**	18.4	18	17.8	**17**
$Net_{2}$	110	15.9	**15**	20.9	18	20.9	20	15.5	**15**	16.4	**15**
$Net_{2}$	120	13.8	13	14.6	14	14.4	13	15.2	14	13.2	**12**
$Net_{3}$	70	46.5	45	37.3	**36**	38.9	37	36.5	**36**	37.6	**36**
$Net_{3}$	80	37.5	**35**	37.4	**35**	39.5	37	36.4	36	35.6	**35**
$Net_{3}$	90	30.9	**30**	34.9	34	35.9	35	34.5	33	34.3	32
$Net_{3}$	100	25.8	25	31.4	29	33.4	30	30.8	28	25.9	**22**
$Net_{3}$	110	22.7	**22**	30.0	28	29.4	28	29.7	28	29.2	27
$Net_{3}$	120	19.1	**18**	27.8	27	27.8	25	27.4	27	27.8	27
$Net_{4}$	100	46.5	45	34.4	33	35.2	34	35.2	35	34.2	**32**
$Net_{4}$	110	39.5	**37**	40.6	39	40.3	38	39.1	38	38.7	**37**
$Net_{4}$	120	35.4	**34**	34.8	**34**	35.9	35	36.2	36	35.9	**34**
$Net_{4}$	130	30.5	**29**	35.8	35	37.8	36	36.5	36	36.1	35
$Net_{4}$	140	34.3	**25**	33.6	33	35.3	34	32.6	32	33.6	32
$Net_{4}$	150	24.3	**23**	30.8	29	33.8	30	32.2	30	29.2	29
$Net_{4}$	160	22.3	**22**	30.8	30	30.9	28	31.2	30	31.6	30
$Net_{5}$	130	85.6	57	48.6	**43**	48.2	46	46.4	45	44.0	44
$Net_{5}$	140	52.3	50	50.5	47	49.8	49	47.2	**46**	47.2	**46**
$Net_{5}$	150	48.5	46	44.2	43	45.9	**35**	44.6	44	44.5	43
$Net_{5}$	160	43.7	43	41.6	41	42.8	41	42.3	41	41.3	**40**
$Net_{6}$	200	50.4	49	61.5	59	63.6	56	48.6	46	46.7	**45**
$Net_{6}$	210	46.1	45	46.8	43	47.9	45	46.8	46	44.2	**43**
$Net_{6}$	220	42.1	**40**	49.2	48	49.1	47	48.3	47	47.4	46
$Net_{6}$	230	39.8	**39**	44.9	43	46.8	45	44.9	44	43.2	42
$Net_{7}$	200	75.4	73	56.2	54	58.5	57	55.6	55	55.4	**53**
$Net_{7}$	210	70.0	67	69.2	**64**	67.9	66	66.1	65	65.4	**64**
$Net_{7}$	220	67.0	62	64.4	**61**	64.5	62	62.3	**61**	61.9	**61**
$Net_{7}$	230	60.9	59	57.7	55	55.6	55	54.6	**54**	55.6	**54**
$Net_{8}$	210	94.7	90	75.0	**70**	75.5	**70**	72.6	71	71.6	**70**
$Net_{8}$	220	87.9	82	77.3	**73**	79.8	74	74.2	**73**	74.5	**73**
$Net_{8}$	230	81.6	78	70.8	69	72.1	71	70.6	69	70.7	**68**
$Net_{8}$	240	76.1	74	68.0	**65**	72.3	67	70.2	67	71.5	**65**
Overall Average		39.2	36.8	37.6	35.7	38.5	36.0	36.6	35.5	35.9	**34.3**
No. of Beats		27	24	33	24	37	35	30	28	–	–
